# Design of Cone Penetration Test Data Relay Transmission by Magnetic Communication

**DOI:** 10.3390/s22134777

**Published:** 2022-06-24

**Authors:** Xiuxin Yu, Shuting Liu, Hongxing Pei

**Affiliations:** 1 School of Physics, Zhengzhou University, Zhengzhou 450001, China; 202022132012383@gs.zzu.edu.cn (X.Y.); jwphx@zzu.edu.cn (S.L.); 2 Henan Academy of Big Data, Zhengzhou University, Zhengzhou 450052, China

**Keywords:** CPT, magnetic communication, cableless, underground sensors, data relay

## Abstract

At present, most cone penetration test experiments use cables to transmit data. The cables not only make the exploration operation very complicated, but also hinder the realization of automatic exploration. An excessively long cable can also bring about additional attenuation and noise during the transmission of probe signal. In order to simplify the procedures of exploration operation and to improve the detection accuracy, a cableless cone penetration test system is proposed in this study. It improved the system by using magnetic communication and converts the electrical signal into a magnetic signal at the connection of two adjacent probe rods for relay transmission. Exploratory experiments were carried out to evaluate the feasibility and accuracy of the new system. The experimental results show that the experimental data collected by the new system is more accurate than that collected by traditional CPT equipment with cable. The new system simplifies exploration operations and enables real-time data transmission to detect abnormalities in time. This anomaly usually means that the probe is pressed against hard rock. It is more convenient and accurate to use the new system for exploration.

## 1. Introduction

Cone penetration test (CPT) refers to a process that drives the probe with sensors into the soil through moderate and stable pressure at a constant speed as far as possible and measures the stress from the probe tip and the friction from the side wall in this process. After analyzing the stress from the probe at different depths by means of statistics, we can infer the properties of a soil layer [1,2,3]. CPT is characterized by a lower cost but higher reliability. It is widely used in geological exploration, building foundation monitoring, constructing offshore platform, evaluating soil condition, and so on [4,5,6,7,8].

**Figure 1:**
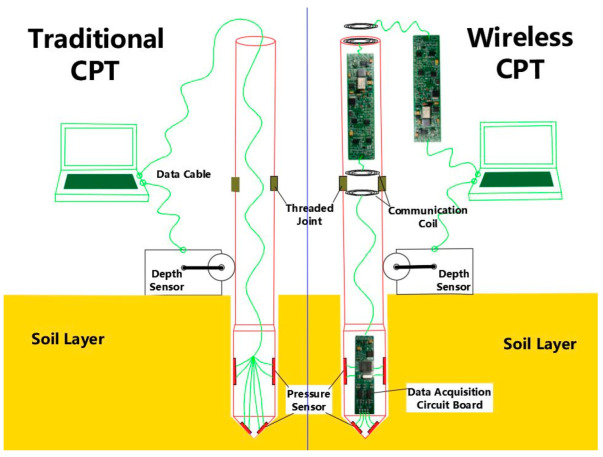
Schematic diagram of static cone penetration test.

The traditional CPT device is shown in the left part of Figure 1. Steel pipes are connected by threads to form a CPT string. On the end of the CPT string is an electronic probe with a conical tip, and various sensors can be installed inside the probe. The CPT string is usually pushed vertically into the soil at a speed of about 2 cm/s by a hydraulic thrust device [9]. When the CPT string moves into soil, the roller of the depth sensor coupled with the string through friction will rotate. A photoelectric rotating encoder set in the depth sensor will record the depth of the probe under the ground when the probe is moving. The signal collected by the probe is transmitted to the receiving equipment on the ground in real time through a long cable passed through all the probe rods [10]. The receiving equipment will amplify, digitize, analyze and store the signal in turn. The receiving equipment will display the final pressure data on the screen for the ground operator to view. If operators find abnormal data and can judge that the probe hit the hard rock stratum, they need to stop in time to avoid damage to the probe.

The probe of some more advanced CPT equipment is also equipped with a sensor for measuring the inclination angle, which can measure the inclination angle of CPT string. When the CPT string is seriously inclined, the pressure data will not match the depth, and even the CPT string will be broken [11]. Therefore, in this case, it is also necessary to stop prospecting in time. The existence of cable in traditional CPT has seriously increased the difficulty of probe rod connection, making it impossible to use machinery to realize automatic connection and continuous penetration. Moreover, a long cable will cause noise and distortion in the process of signal transmission. When the cable is too long, the transmitted signal can no longer be trusted.

**Figure 2:**
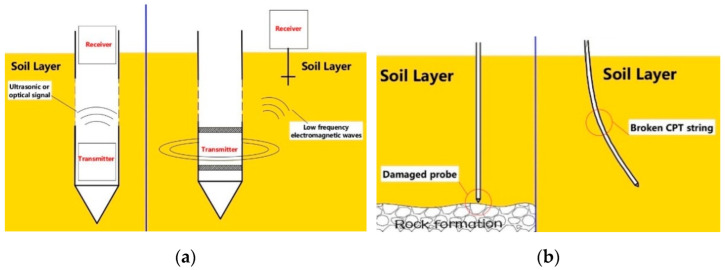
Some new methods and special cases: (**a**) optical communication and electromagnetic wave communication; (**b**) the probe is squeezed by the rock or the CPT string is bent.

Therefore, the present study is engaged to develop a cableless system, which can transmit data from the detector deep underground to the receiving equipment on the ground in real time. Currently, there are some technologies that can meet such requirements [12]. It mainly uses ultrasonic or optical communication, underground radio and probe rod with communication function [13,14,15,16,17]. Because the actual exploration conditions are very chaotic, it is difficult to ensure that the inside of the probe rod is clean, and it is also difficult for the metal threaded joint to prevent the infiltration of mud and water in the soil layer with high water content. Using ultrasonic or optical communication, if sewage or sludge is mixed in the pipeline, the communication will be interrupted. When using underground radio, its stability is easily affected by soil properties, such as soil conductivity, moisture content and temperature [18,19,20]. Moreover, high-frequency electromagnetic waves attenuate very quickly when propagating underground, so only electromagnetic waves with extremely low frequencies can be served as the carrier, which results in a very low data transmission speed. When the exploration depth increases, the signal transmission power also needs to increase sharply, which undoubtedly will reduce the working time of the device. Figure 2a show some existing methods.

The use of special probe rods with communication function seems to be a feasible method. However, it has not been promoted yet, which possibly because it is extremely difficult to maintain these fine structure steel pipes in harsh exploration environment. Other researchers try to temporarily store the exploration data in the probe underground and take out the data in the probe after the exploration. Although CPT does not require high real-time data, it is unacceptable in most cases if the underground state cannot be known in the whole exploration process. When the probe is pressed onto the hard rock stratum and cannot stop in time, it will probably damage the probe and lose data [21]. When the CPT string is seriously inclined, the pressure data will not match the depth, and even the CPT string will be broken. All these require that the state of the underground probe can be known during the exploration process. Figure 2b shows these situations.

These new wireless technologies are all attempts to send sensor data to the receiver at one time. However, both the environment inside the steel pipe and the soil environment outside the steel pipe are complex, the long-distance transmission is easy to cause information loss or distortion. In order to solve this problem, the data collected by the sensor can be transmitted to the receiving device by means of relay. When the transmission distance is short enough, the reliability of communication can be guaranteed. Based on this, we designed a new system. The proposed system uses magnetic communication technology to convert electrical signals into magnetic signals for relay transmission at the junction of two adjacent probe rods.

## 2. System Design

The new CPT system includes a probe, pressure sensor, depth sensor, data acquisition circuit, data relay circuit and control program. The right half of Figure 1 shows the structure of the new system. The pressure sensor is embedded in the probe, and the data acquisition circuit board and battery are installed in the probe lumen. To prevent water ingress when working underground, the magnetic communication coil, data relay circuit board and battery are sealed in a plastic pipe and then plugged into the CPT metal probe rod. Each probe rod is equipped with batteries, receiving and transmitting circuits, which can receive and retransmit data.

**Figure 3:**
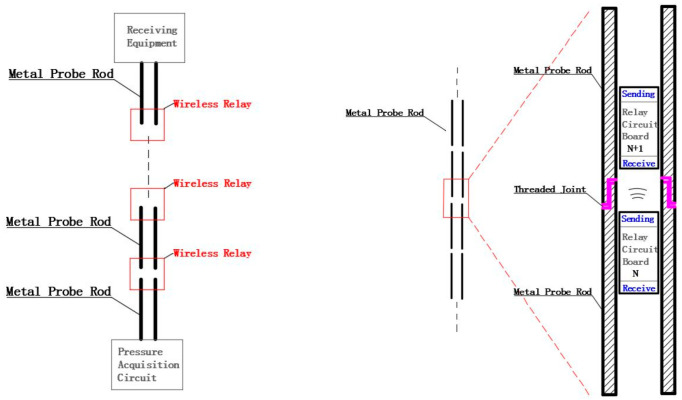
The diagram of the signal transmission process of the proposed system.

Figure 3 is the schematic diagram of system signal transmission. The data acquisition circuit board captures probe tip stress and sidewall friction as the hydraulic thruster presses the probe into the ground. The MCU modulates the obtained pressure data and transmits it to the data relay circuit in the first probe rod connected to the probe through magnetic communication. The first relay circuit demodulates the magnetic signal to obtain data, and then modulates the data again and transmits it to the relay circuit in the next adjacent probe rod. In this way, the data will be transmitted upward in turn, and finally transmitted to the receiving equipment on the ground. On the one hand, the receiving device receives the pressure data from the probe, and on the other hand, it obtains the current depth through the depth sensor. The system can realize real-time data transmission and avoid many problems caused by the traditional way of using long cable to transmit data.

In order to verify the feasibility of the design scheme, we produced a prototype and conducted field exploration tests. The test results showed that the experimental data collected by the new system are more accurate than the data collected by traditional CPT equipment. The communication circuit is placed inside the metal probe rod, and there is a plastic waterproof seal to protect it. Therefore, it is usually not necessary to consider the influence of soil conditions (such as moisture content, conductivity, hardness, temperature) on the system. The new system simplified exploration operations and enabled reliable real-time data transmission.

## 3. Design of Pressure Acquisition Circuit

**Figure 4:**
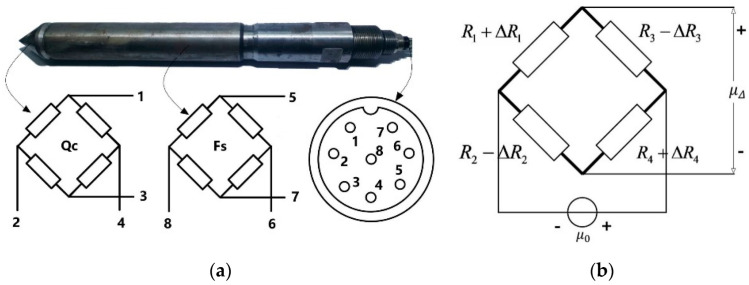
Sensor layout and electrical interface of the CPT probe. (**a**) sensor location and interface; (**b**) bridge circuit diagram.

We used a common standard probe whose actuality is shown in Figure 4a. It does not need to be specially made, you can buy it easily. The cylindrical probe has a conical tip of apex angle equal to 60° and a bottom area of 15 cm^2^, the surface area of side wall friction cylinder is 300 cm^2^. The probe tip can withstand a maximum force of 6300 N. Two Wheatstone bridge sensors are set inside the probe. The pressure sensor is composed of four metal foil resistance strain gauges. When the strain gauge is bent by external force, the resistance value changes and the bridge loses balance to produce voltage output [22,23].

The two bridges are very similar, so we take one of them as an example; its structure is shown in Figure 4b. The relationship between its output voltage $\mu_{\Delta }$ and the four resistors is as follows:

(1) $$\mu_{\Delta }=\mu_{0\;}\left(\frac{R_{1}+\Delta R_{1}}{R_{1}+\Delta R_{1}+R_{3}-\Delta R_{3}}-\frac{R_{2}-\Delta R_{2}}{R_{4}+\Delta R_{4}+R_{2}-\Delta R_{2}}\right)$$

In the probe we used, the four strain gauge resistance structures of the bridge are identical. The resistance *R* of the four strain gages in the static state without deformation is equal, and they are all 1 kilo-ohm. In fact, the four strain gauges are placed symmetrically in the probe. At the same moment, their resistance value changes ΔR can also be approximately considered to be the same, which means:$\;\Delta R_{1}\;$=$\;\Delta R_{2}\;$=$\;\Delta R_{3}\;$=$\;\Delta R_{4}\;$= ΔR. So we can get the following relationship:

(2) $$\mu_{\Delta }=\mu_{0\;}\left(\frac{R+\Delta R}{R+\Delta R+R-\Delta R}-\frac{R-\Delta R}{R+\Delta R+R-\Delta R}\right)=\mu_{0\;}\frac{\Delta R}{R}$$

**Figure 5:**
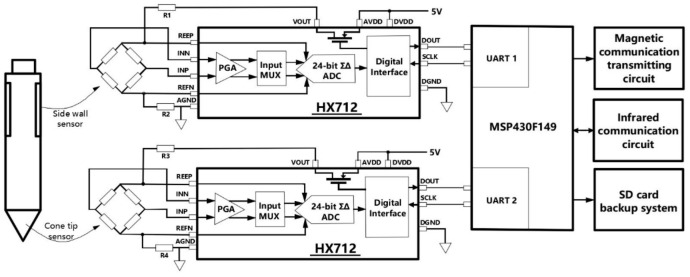
Circuit diagram of the data collector in the CPT probe.

Applying different pressure to the probe, the value of ΔR will be different, so different $\mu_{\Delta }$ can be obtained. Based on this, we measured a series of values in the laboratory and calibrated the probe. Two HX712 chips are used to process two bridges, respectively. HX712 is a dedicated chip for measuring bridge sensors, it has a low noise amplifier with a gain of 128 times and a 24-bit high precision ADC. When the chip is in standby, the MOS switch in the chip will turn off the power supply of the sensor. According to the data provided by the manufacturer, the standby current of HX712 is less than 1 µA, and the typical operating current is 1 mA [24]. The ultra-low power consumption is beneficial for prolonging the time of using the probe underground. The MCU of the acquisition circuit uses MSP430F149 produced by Texas Instruments (Dallas, TX, USA), which can enter low power mode when it is not working [25,26]. MSP430 Series MCU is famous for its low power consumption. The composition of the acquisition circuit is shown in Figure 5.

For each HX712, the MCU continuously accesses five times per second to obtain the pressure value detected by the two sensors in the probe. The MCU will take the average of the last four times as the current pressure. These two pressure values will be modulated by the MCU together with the current system time and will be sent to the relay circuit in the probe rod through the transmitter circuit. The specific modulation method and transmitting circuit will be introduced in the next section. To increase system reliability, data is also backed up to a SD memory card. In extremely special cases, when the relay communication cannot be carried out normally, the SD card can be taken out after the exploration to obtain exploration data.

## 4. Magnetic Communication Repeater Design

**Figure 6:**
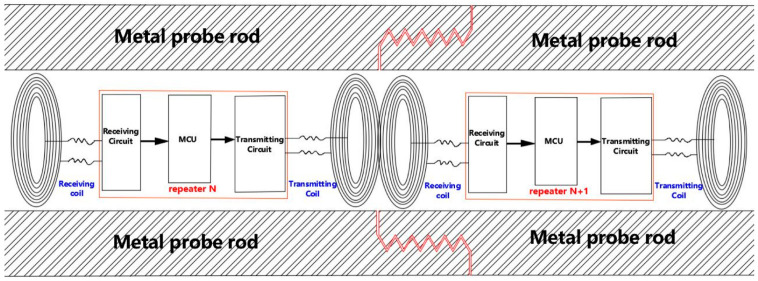
The composition of the repeater and the pairing method of the coil.

The repeater is composed of signal transmitting circuit, signal receiving circuit, MCU, communication coil, power management circuit and battery. Figure 6 shows the composition of the repeater and the pairing of the coils. During exploration, it is necessary to connect the metal probe through the threaded joint, and the communication coils will be close to each other during the process of tightening the threaded joint. After the threaded joint is connected, the distance between the paired transmitting coil and the receiving coil is usually less than 2 cm. During the test, we set the distance to 6 cm and could still communicate normally. Obviously, a smaller distance under actual working conditions is better to ensure the quality and stability of communication.

### 4.1. Modulation Method and Transmitting Circuit

**Figure 7:**
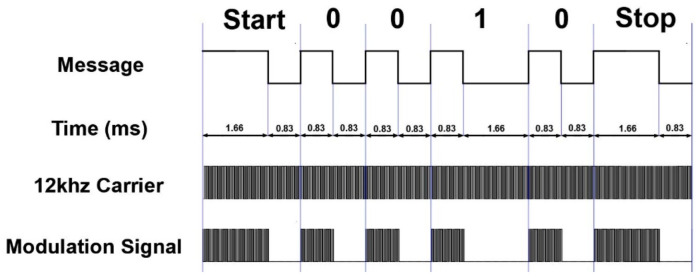
The composition and generation process of the modulated signal.

We use digital signals modulated by pulse width to transmit information [27], and the information encoding method is shown in Figure 7. A combination of a high electric level of 0.83 ms and a low electric level of 1.66 ms is representative of binary 1. A combination of a high electric level of 0.83 ms and a low electric level of 0.83 ms is representative of binary 0. In order to indicate the start and the end of data transmission, a start code and an end code need to be added before and after each transmission data. We can make the start and end code the same to reduce the complexity of programming. Therefore, both are composed of 1.66 ms high electric level and 0.83 ms low electric level.

In extreme cases, if the transmitted data is all binary 1, the communication speed of the system is the slowest. We can calculate the transmission speed $Rat_{min}$ at this time, as shown in Equation (3). Obviously, the minimum communication speed of the system can reach 400 bit/s, which is completely sufficient for transmitting the values of two sensors in the CPT probe. If several sensors (such as sensors for measuring tilt angle) are added in the probe, this speed is also sufficient.

(3) $$Rat_{min}=\frac{1000}{0.83+1.66}=401.6\;\;\mathrm{bit}/s$$

In order to reduce the system cost, STC15F104 is selected as the MCU of the relay circuit, which has limited performance and cannot handle signals with high frequency. At the same time, the high frequency signal attenuates quickly in the metal pipe [28,29], which also requires the use of a lower carrier frequency. So, we set the carrier frequency to 12 kHz, which is a very low frequency. The information is loaded onto a square carrier with a frequency of 12 kHz and a duty cycle of 33% to form a modulated signal.

**Figure 8:**
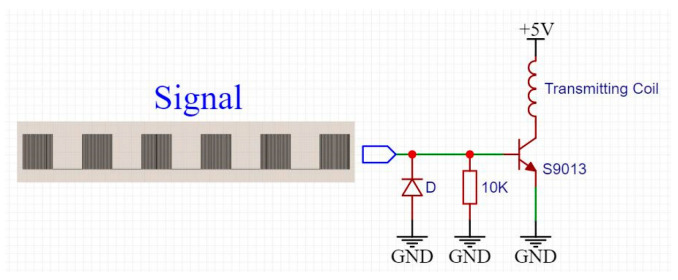
Test signal and transmitting circuit.

A simple transmitter circuit is shown in Figure 8. The MCU of the relay circuit uses it to transmit modulated signals. The transmitting coil is placed on the position shown in the figure; the MCU loads the modulation signal on the base of the transistor. When we design the receiving circuit in Section 4.2, the ‘0’ signal was loaded as the test signal: the square wave lasted 0.83 ms, and the no square wave lasted 0.83 ms, and these two states were executed alternately.

### 4.2. Signal Receiving Circuit Design

#### 4.2.1. Signal Amplifier Circuit Design

**Figure 9:**
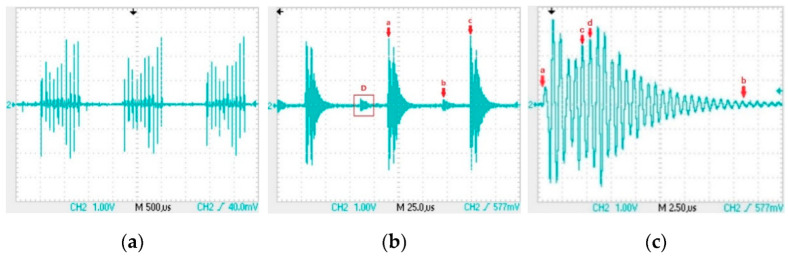
The waveform of the signal in the receiving coil: (**a**) signal waveform; (**b**) induced signal generated by continuous square wave in transmit coil; (**c**) oscillation signal generated by falling edge of square wave in transmitting coil.

We load the “0” signal into the transmitting circuit and use a receiving coil to capture the signal. Analyze the signal waveform in the receiving coil to design a suitable signal amplification circuit. Figure 9 is the signal waveform received by the receiving coil, which is sequentially amplified as shown in the following three figures. In Figure 9b, the duration from point a to point c is 83 µs; the duration from point a to point b is 56 µs; and the duration from point b to point c is 27 µs. The duty cycle of the 12 kHz carrier loaded in the transmitting coil is 33%. It can be seen that the inductive signal in the receiving coil is mainly generated by the falling edge of the carrier wave in the transmitting coil, and the inductive signal caused by the rising edge is very small. The signal in the red D box in Figure 9b is caused by the rising edge. Enlarging a voltage fluctuation generated by a falling edge in Figure 9b to obtain Figure 9c, the total duration of the signal from point a to point b in Figure 9c is 20 µs, and the duration from point c to point d is 700 ns.

**Figure 10:**
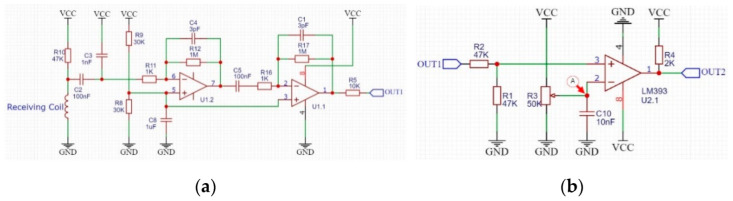
Signal amplifier circuit and first comparator circuit: (**a**) signal amplifier circuit and its output: OUT1; (**b**) first comparator circuit and its output: OUT2.

In order to obtain the transmitted information from the signal of the receiving coil, we first restore the signal to a modulated signal similar to that shown in Figure 7, and then remove the carrier in the modulated signal, and finally obtain the transmitted information. If software is used to implement these processes, it will demand high performance of MCU, which will inevitably lead to an increase in cost, especially when each relay circuit needs a piece of such MCU. Therefore, we choose to use hardware circuit to obtain information we need. First, an amplifier circuit is used to amplify the weak signal in the receiving coil. Since digital signals are used to transmit information, it is not necessary for the amplifying circuit to strictly maintain the waveform of the original signal on the receiving coil during the process of amplifying the signal. We use a capacitor to obtain the average value of each signal and process each induced signal similar to Figure 9c as a square wave. The designed signal amplification circuit is shown in Figure 10a. The power supply in the figure uses two 3.7 V lithium batteries in series.

**Figure 11:**
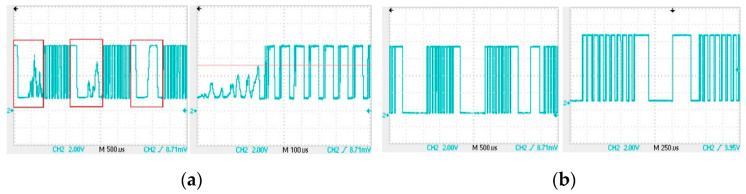
Waveform diagram of OUT1 signal and OUT2 signal: (**a**) output OUT1 of the amplifier circuit; (**b**) output OUT2 of the first comparator.

In order to deal with these weak signals, for the operational amplifier in this article, we have used JFET-input amplifiers [30,31], model OPA2604, which has higher input impedance than the generally used ones. Connect the receiving coil to the amplifier circuit shown in Figure 10a, the waveform of the output signal OUT1 is shown in Figure 11a. The signal shown in red box of Figure 11a is the output when the input is low, and output should also be low in theory. Obviously, we can see noise in the red box. The noise here can be divided into two parts: the first part of noise has a similar frequency to the signal but a lower peak voltage, while the second part of noise has a similar peak voltage to the signal but with significantly different frequencies. A comparator as shown in Figure 10b can be used to filter out first parts of noise with lower peak voltages. Adjust the R3 resistor in Figure 10b so that the voltage at point A in the figure is about 5 V, which is the voltage represented by the red horizontal line in Figure 11a. In this setting, the voltage comparator can remove noise with lower peak voltage, and its output is OUT2, and the waveform is shown in Figure 11b.

#### 4.2.2. Signal Filter Circuit

**Figure 12:**
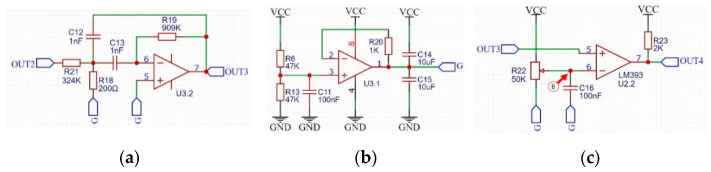
Signal processing circuit: (**a**) filter circuit and its output: OUT3; (**b**) analog ground voltage generation circuit and its output: G; (**c**) second comparator circuit and its output: OUT4.

By observing the output OUT2 of the voltage comparator, we used a bandpass filter to remove the second part of noise. The filter circuit we designed, shown as Figure 12a, is a Chebyshev bandpass filter with a center frequency of 12 kHz and a bandwidth of 500 Hz. Figure 12b is a voltage follower that obtains half of the supply voltage through a resistor divider. This voltage is used as the analog power ground G. It is supplied to the filter and the voltage comparator in Figure 12c.

**Figure 13:**
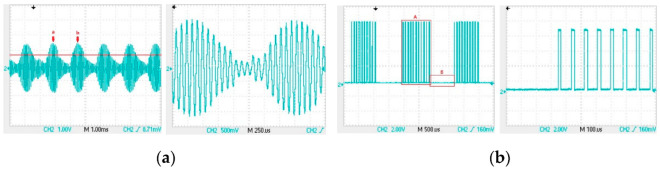
Waveform diagram of OUT3 signal and OUT4 signal: (**a**) filter output OUT3; (**b**) output OUT4 of the second comparator.

We got the output OUT3 after connecting the OUT2 signal to the filter. Figure 13a is the signal waveform between OUT3 and the analog power ground Ga. The period of the signal in Figure 13a is 83 µs, and the time interval between point a and point b is 1.66 ms. There is no noise between point a and point b, thus a voltage comparator can be used to take out the signal from OUT3. Connect OUT3 to the voltage comparator shown in Figure 12c is to obtain the output OUT4. Adjusting the R22 resistor in Figure 12c, so that the voltage at point B is about 0.7 V, which is the voltage represented by the red horizontal line in Figure 13a. Such an adjustment can make the duration of the square wave in the output OUT4 of the voltage comparator and the duration of no square wave roughly equal, and both durations are 0.83 ms. Figure 13b is the waveform of the output OUT4 of the voltage comparator, and it can be seen that the signal has been processed into a modulation signal similar to that in Figure 7.

#### 4.2.3. Signal Reduction Circuit Design

Comparing the output OUT4 waveform of the filter in Figure 13b with the output OUT1 waveform of the amplifier circuit in Figure 11a, it can be seen that the noise has been removed in OUT4. Now it is only necessary to process the square wave in the OUT4 signal as a high level, so that the carrier wave can be removed to obtain the information waveform in Figure 7.

**Figure 14:**
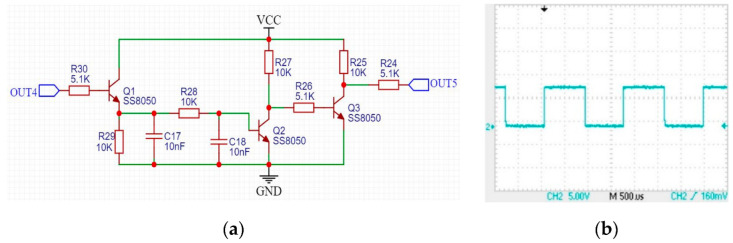
The integrator circuit and its output OUT5: (**a**) integrator circuit (**b**) output OUT5 of the integrator circuit.

It can be noticed that the duration of the square wave of OUT4 in Figure 13b is 22 µs and 62 µs separately when it is in high and low voltage, and the duty cycle is about 26%. However, the duty cycle of the 12 kHz carrier initially loaded on the transmitting coil is 33%. The different duty cycle is because the signal in the receiving coil is only related to the level mutation of the signal in the transmitting coil but has nothing to do with its duty cycle. We adjusted the duty cycle of the carrier in the transmitting coil to 60%, and the duty cycle of the square wave in OUT4 is still about 26%. Fortunately, the square wave in the OUT4 will eventually be processed as a high-voltage signal, and the duty cycle did not affect this process. We used an integrating circuit shown in Figure 14a to remove the carrier wave. We connected the output OUT4 of the filter to this circuit. When there was a continuous square wave in OUT4 (box A in Figure 13b), OUT5 was high-voltage signal. When there was no continuous square wave in OUT4 (box B in Figure 13b), OUT5 was low. The waveform of the output OUT5 of the integrating circuit is shown in Figure 14b.

After this series of circuit processing, the period of the final output signal OUT5 is about 1.66 ms, and the duty cycle is about 50%. The waveform of OUT5 is basically the same as the 0 signal in Figure 7. Although the duty cycle of OUT5 is affected by the adjustable resistor R22 in Figure 12c, generally speaking, when R22 is properly adjusted and the receiving program allows some time error in waveform pulse width, the duty cycle error does not affect the correct transmission of information.

**Figure 15:**
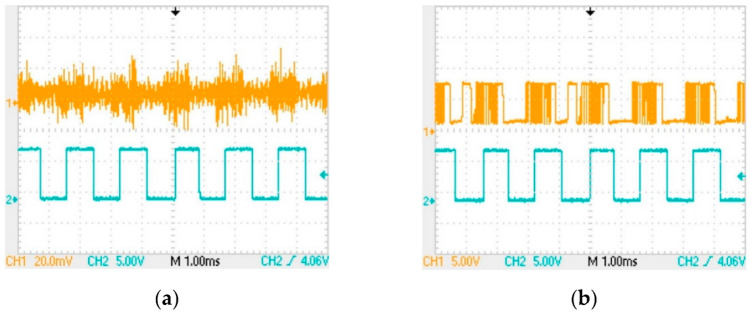
Signal comparison diagram: (**a**) comparison of the OUT5 signal with the signal in the receiving coil; (**b**) the comparison between the OUT5 signal and the output OUT1 signal of the amplifier circuit.

The CH1 channel in Figure 15a is the original signal waveform in the receiving coil (the coil has been connected to the circuit); the CH1 channel in Figure 15b is the output signal OUT1 of the amplifier circuit; and the CH2 channel in both figures is the waveform of the final signal OUT5. As can be seen from Figure 15, the final output signal has a delay of about 0.3 ms compared with the original signal in the receiving coil.

### 4.3. MCU and Auxiliary Circuit

The signal OUT4 in the receiving circuit is very similar to the modulated signal in Figure 7. We load OUT4 directly on the transmitting circuit in Figure 8, and the information can be transmitted to the next relay circuit through the communication coil. However, this approach has a problem of accumulating errors. In the actual test, after 6 relay transmissions in this way, the output OUT5 of the 7th receiver at the end is observed. The high electrical level duration of this OUT5 signal exceeds 1.1 ms (the normal value should be 0.83 ms), and the duty cycle reaches 66%. Such errors are unacceptable and will become more uncontrollable as the number of relay transmissions increases.

**Figure 16:**
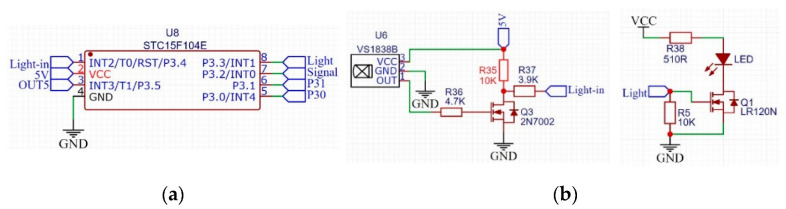
MCU and infrared communication circuit: (**a**) STC15 microcontroller circuit; (**b**) infrared communication circuit.

**Figure 17:**
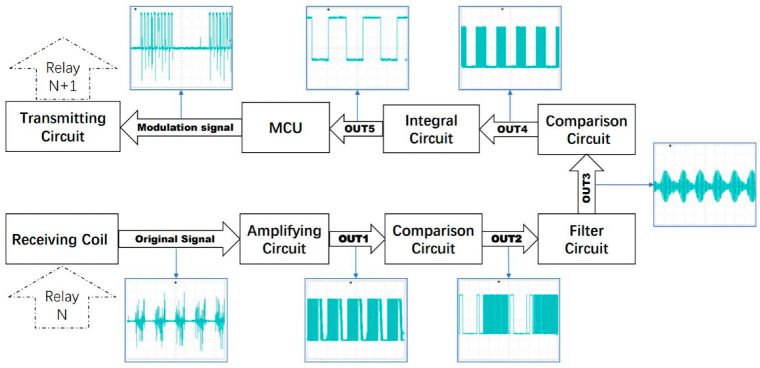
Signal processing flow chart.

To eliminate accumulated errors, we ordered each repeater circuit re-modulated the information once. We connected the output OUT5 of the receiving circuit to a MCU, and the MCU would re-modulated the information after getting the information. Finally, the modulated information was loaded into the transmitting circuit to transmit it to the next relay circuit. In this way, the signal transmitted by each relay circuit was re-modulated by the MCU, by which the error could be eliminated. Each probe rod needs a relay circuit, which means a large amount of MCU is needed as well. Therefore, the cost of a single MCU should be reduced as much as possible to control the cost of the whole system. We chose STC15F104 produced by STC company [32], and its circuit is shown in Figure 16a. We connected the signal OUT5 output by the receiving circuit with the P3.5 pin of STC15F104 to receive the signal and connected the base of the transistor in the transmitting circuit in Figure 8 with the P3.2 pin of STC15F104 to send the modulated signal. Thus, we designed the relay circuit, and its signal processing flow is shown in Figure 17.

We also added an infrared communication circuit on the basis of the above design, and the circuit is shown in Figure 16b. The modulation mode of infrared communication is the same as that of magnetic communication in Figure 7. However, the only difference is that its carrier frequency we used is 38 kHz [33,34]. Although in actual working conditions, there is often sludge at the joint of the probe rod, which leads to the failure of infrared communication. However, the cost of adding the infrared communication circuit is very low. In special cases, when the magnetic communication fails, another communication method can be provided to communicate. Such a design can improve system redundancy and increase reliability.

There is another important reason to add infrared communications. The receiver on the ground in Figure 1 is directly connected to the computer by a data cable. The receiver can receive information through magnetic communication or an alternate infrared communication, but the distance of magnetic communication is very limited. The longest distance that can guarantee normal communication in the actual test is about 10 cm. If magnetic communication is used, the receiver needs to follow the rod down as it moves down. We actually fixed the receiver directly above the CPT string. When magnetic communication fails, the receiver automatically switches to infrared communication. Infrared communication can be up to tens of meters, so the receiver can be fixed. The receiver can be at least a few meters away from the end of the CPT string, which will allow the operation of connecting the new rod to be unimpeded.

**Figure 18:**
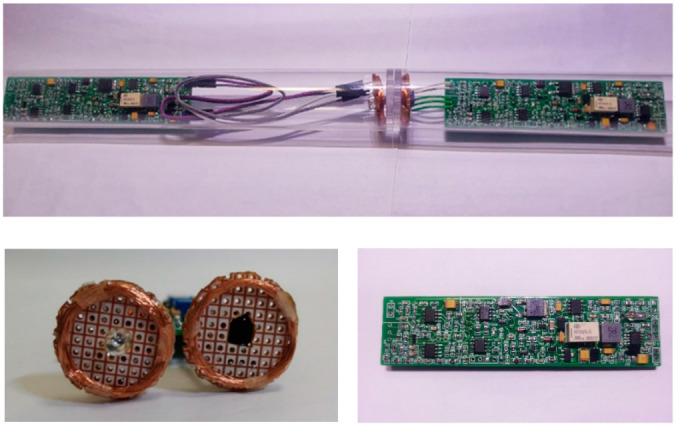
Image showing the relay node.

The physical circuit of a basic relay node is shown in Figure 18. The circuit board, battery and coil were encapsulated together in a plastic tube and tucked into the CPT metal probe as a unit.

## 5. Power Management Circuit Design

**Figure 19:**
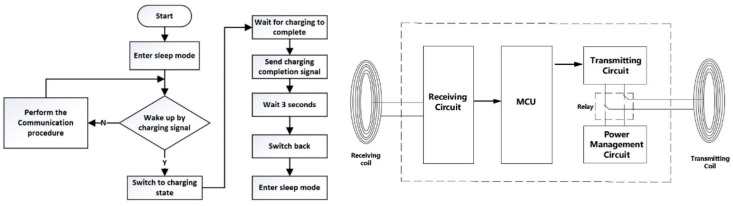
Power management logic.

For the convenience of use and maintenance, we set up the gravity switch and wireless charging circuit in the power management circuit, which avoids to opening the watertight plastic tube when charging or switching on power. Figure 19 shows the logic of power management. The power supply of the system uses two 3.7 V lithium batteries in series. Gravity switches were set on the acquisition circuit board and relay circuit board. When the probe rod or probe is placed upright, the gravity switch will be turned on, and the circuit board will be automatically energized. When they were put flat in the process of storage and transportation, the power supply will be closed.

During wireless charging, the signal transmitting coil in magnetic communication is used as the power receiving coil, and the micro relay HFD4 is used to switch the two states of the coil. The CPT probe rod or probe that needed to be charged was placed upright on the charging stand, and the gravity switch would be turned on. The charging stand will send a charging signal to the device needed to charge through infrared communication. After the MCU of the device that needs to be charged receives the signal, it energized the coil of the relay HFD4 to switch the transmitting coil of the magnetic communication to the charging circuit so that it can turn into the power receiving coil, and feedback the ready completion signal to the charging stand. After a few seconds, the charging stand will charge the device through this coil. When the charging is completed, the MCU send the end signal of charging to the charging stand through infrared communication; The charging stand will stop wireless charging immediately, and after a few seconds relay HFD4 will switch the transmitter coil back to the transmitter circuit of the magnetic communication. At this point, the charging is completed, and the device enters the standby state. It can be removed from the charging base and stored horizontally or upside down, waiting to be used during exploration.

Such a power management circuit contributes to the communication system no longer needs to be taken out from the probe rod after it is installed, which greatly facilitates the use and maintenance of the equipment.

## 6. Control Program

The circuit board of pressure data acquisition in the CPT probe is required to realize the functions of collecting analog signals, modulating information, transmitting magnetic communication signals and recording system time. When the probe touches the ground, the acquisition circuit board in it will detect the pressure and send a start signal to the receiving device on the ground. At this time, the circuit board of pressure acquisition in the probe and the receiving device on the ground will start timing from 00:00:00 at the same time. Then, the pressure acquisition circuit collects a set of data per second as the hydraulic thrust device pushes the probe into the ground. When not working, the MCU will enter sleep mode to reduce power consumption [35]. After the sleep is over, the first step is for the MCU to access the two HX712 chips five times to obtain the values of the tip and sidewall bridges, respectively, and take the average of values collected in the last four times. After the access is over, HX712 will automatically stop supplying power to the sensor bridge, and HX712 itself will also go into sleep and wait for the next second MCU access to it. The second step is that the MCU modulates these two average values together with the current system time according to the method described in Section 4.1 and loads the modulated signal on the transmitting circuit to transmit these data. The third step is to back up the sent data to the SD card to ensure that in the extreme case of real-time communication interruption, the data can still be obtained from the SD card in the probe after the exploration is over. The fourth step is that the MCU enters the low power mode and waits for the end of the one second timer, and then performs the next work cycle.

The relay circuit board in the CPT probe rod needs to realize the functions of receiving communication signals, modulating information and transmitting communication signals. The first step is that the relay circuit in the first probe rod adjacent to the probe receives the magnetic communication signal from the probe and obtains the information through the receiving circuit. Then, the information will be re-modulated by the MCU in the relay circuit and sent out in two ways, magnetic communication and infrared communication. The second step is that the relay circuit in the second probe rod receives the information from the first probe rod, modulates it and transmits it again by magnetic communication and infrared communication. If the relay circuit dose not receive a message from the previous probe for more than two seconds, it will attempt to receive the message using infrared communication. The third step is to sequentially transmit the information to the ground in this way until the information is received by the receiving equipment on the ground. Ground receiving equipment consists of a receiver and a computer. The receiver is identical to the relay circuit board, except that it does not use infrared or magnetic communication transmission functions, but directly transmits data to the computer through the data cable.

Either the acquisition circuit or the relay circuit will perform the charging procedure described in Section 5 when they are in the charging state. During exploration, the computer on the ground receives the data from the probe and also perceives the current extract depth of the probe through the depth sensor. Under normal circumstances, the data transmitted from the probe will be directly displayed and stored by the computer. However, when the detection depth is very deep, it will take too long to transmit the data to the computer on the ground. At this time, the pressure data of the probe will lag behind the depth, and the two will not match.

**Figure 20:**
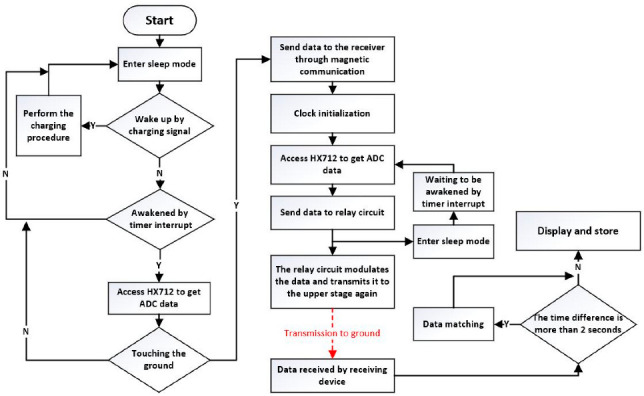
Program flow chart.

Therefore, we set up an error correction mechanism. When the time contained in the information transmitted from the probe lags behind the time of the computer on the ground for more than 2 s, the depth data and the pressure data of the probe will be paired through time. The downward driving speed of CPT is usually about 2 cm/s, and the exploration depth error caused by the delay of 2 s will not exceed 4 cm, so the small error is within the allowable range. Figure 20 shows the program flow of the system.

## 7. Exploration Testing and Results

**Figure 21:**
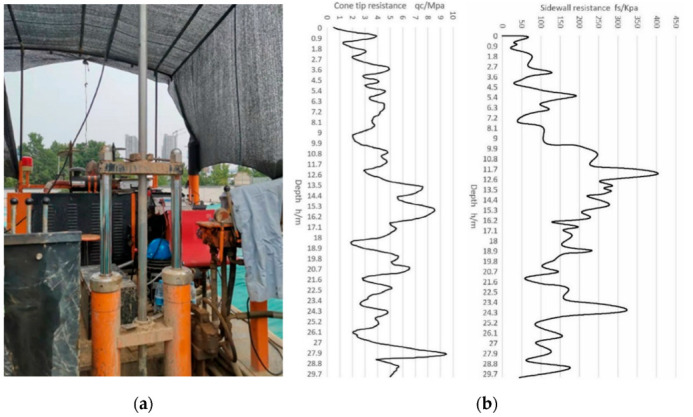
Experimental equipment and experimental data sheets: (**a**) hydraulic thrust device; (**b**) the curve diagram of the stress that the probe receives.

The cableless CPT system proposed in this paper was calibrated in the laboratory after a prototype was made in May 2021. After that, we conducted a field exploration test using the prototype. The hydraulic thrust device used in the experiment is shown in Figure 21a, the device was driven by a diesel engine. We applied a vertical downward pressure to the CPT pipe string through the hydraulic machine. According to the previous design, the prototype collects a set of data every second. It took two operators 2.5 h to operate the machine to push the probe into the soil layer to a depth of 30 m. However, this typically requires four operators and takes over 5 h if using the conventional CPT systems.

During the test, the computer on the ground continuously obtains the pressure data from the underground probe and obtains the current depth of the probe through the depth sensor. Through the data analysis software in the computer, the experimental data can be plotted as a curve diagram of probe tip stress and sidewall friction at different depths. We selected 1500 groups of data evenly according to the depth. The graph drawn is shown in Figure 21b. According to the curve, geological researchers can infer the mechanical properties of soil layers at different depths.

By observing the performance of the prototype in the process of use and analyzing the experimental data, we found that there is a certain delay in the transmission of probe pressure data to the ground receiver, but it is usually much less than 2 s. When the probe has an abnormal condition underground, it can be found almost immediately. The new system can measure the probe pressure with an accuracy of 0.01 kPa, which is due to the use of high precision ADC and digital transmission mode. As a comparison, the exploration accuracy of the traditional CPT system model KE-U310 is 0.01 MPa. Data loss rarely occurs during the test, and the communication speed of the new system is fast enough for the data volume of the two bridge CPT. We have tested several sites with significantly different soil layer properties, and the data transmission speed and quality of the new system at the same depth is almost no difference. This suggests that the new system is less affected by the properties of the soil layer.

Due to the failure to prepare more experimental materials, our maximum exploration depth during the experiment is 30 m underground, which is sufficient for common exploration needs. The system uses relays to transmit information, so we expect that increasing the exploration depth will not have too much impact on the normal operation of the system except that it will increase the transmission delay.

## 8. Conclusions

On the basis of traditional CPT, the system uses relay communication instead of traditional cable communication. The new system can work automatically during exploration. The operator only needs to connect the probe rod without having to deal with cables. The communication quality of the new system is hardly affected by the nature of the soil layer and exploration depth. The disadvantage of the new system is that it has some delays. Fortunately, the downward excavation speed of CPT is very slow. Such a small data delay will not affect the normal exploration operation.

Compared with the traditional cabled solution, the up to 24-bit ADC and digital signal transmission method greatly improve the accuracy of the new system exploration. Cableless transmission simplifies the CPT operation process and reduces the time and labor costs of exploration operations. Cableless transmission also makes the design of automated exploration equipment no longer affected by cables, providing the possibility to automate the exploration process in the future. Compared with the existing cableless solution, the system has more advantages in transmission speed, stability and environmental adaptability. Thanks to the specially designed power management system, the maintenance of the new system is also very easy.

## Data Availability

The data that support the findings of this study are available upon reasonable request from the authors.
